# High prevalence of depression and sleep–wake disorders among female emergency medicine residents in South Korea

**DOI:** 10.1080/07853890.2022.2053568

**Published:** 2022-03-29

**Authors:** Mi Jin Lee, Woo Young Nho, Haewon Jung, Jae Wan Cho, Jun Seok Seo, Hyung Min Lee, Kwang Hyun Cho, Yun Jeong Kim, Jong Kun Kim

**Affiliations:** a Department of Emergency Medicine, School of Medicine, Kyungpook National University, Daegu, South Korea; b Department of Emergency Medicine, CHA Gumi Medical Center, CHA University, Gumi, South Korea; c Department of Emergency Medicine, Dongguk University Ilsan Hospital, College of Medicine, Dongguk University, Seoul, South Korea; d Department of Emergency Medicine, Kyung Hee Medical Center, Kyung Hee University, Seoul, South Korea; e Department of Emergency Medicine, Nowon Eulji Medical Center, Eulji University, Seoul, South Korea

**Keywords:** Gender, residents, depression, sleep–wake disorder, emergency medicine, well-being

## Abstract

**Background:**

Depression and sleep–wake disorders are recognized as one of the major problems among emergency physicians. While depression is more common in females than in males, the associated factors linking depression and sleep–wake disorders in emergency physicians, particularly females, remain unknown.

**Objective:**

To analyze the prevalence of depression and sleep–wake problems among emergency medicine (EM) residents in South Korea and to identify the gender differences and situations that adversely predispose female residents to mental health problems.

**Methods:**

We conducted a cross-sectional analysis using the data collected from the 2019 National EM Residents Wellness Survey targeting all of 630 EM residents in South Korea. The survey included variables potentially influencing depression and sleep–wake problems, such as personal characteristics, work-related stress, and extrinsic environment. Information regarding medical conditions, depression, job stress, and sleep deprivation was obtained using the self-administered Patient Health Questionnaire (PHQ-9), the Apgar Wellness Score (AWS), and the Epworth Sleepiness Scale (ESS). We analyzed the data using IBM SPSS Statistics version 25 and MedCalc version 17.

**Results:**

A total of 384 residents participated in the survey. Overall, 27.5% of the EM residents met the criteria for at least moderate depression and 36.9% of the EM residents had sleep-related problems. We found that difficulty in trading the shift schedule and frequent night shifts was associated with depression (*p* = .001, *p* = .005; respectively). Female residents demonstrated an increased risk of depression and sleepiness compared to their male counterparts (odds ratio [OR] 1.95, OR 1.81; respectively). In addition, logistic regression analysis revealed significant differences by gender in depression with regards to flexibility of trading shifts (*p* = .005), level of training in the emergency medical centre (*p* = .035), and frequent night shifts (*p* = .010).

**Conclusions:**

Approximately, one-third of EM residents report depression and sleep–wake problems, with female residents showing a higher risk than male residents. Several risk factors were identified, and future strategies should be aimed to address these issues to improve the training environment and overall wellbeing of EM residents.

**KEY MESSAGES**
- The prevalence of depression and sleep-related problems were, respectively, 1.95 and 1.81 times higher in female residents compared to their male colleagues.
- The associated risk factors for depression were flexibility of shift trade, level of training in the emergency medical centre, and frequency of night shifts.
- Improving the training environment and facilities, as well as offering more flexible duty trading options can provide potential opportunities to reduce the risk.

## Introduction

Depression is a leading cause of disability, and one in 20 people worldwide have experienced an episode of depression [1]. In the medical field, residents are often exposed to numerous stressors, which are strongly associated with symptoms of depression [2]. In this regard, depressed physicians have been shown to be unable to fulfill their professional or personal responsibilities, use more sick days, are at risk of low job performance, and are more liable to commit medical errors, which consequently threatens patient safety [3,4]. In the emergency department (ED), shift work is considered essential, and various types of irregular work schedules are widespread. These irregular work schedules and late-night shifts are known to generally cause sleep deprivation [5]. In addition, studies have shown that the deterioration of the sleep–wake cycle due to sleep deprivation can cause depression [6]. Among medical residents, the prevalence of depression is more frequently reported in females compared to their male counterparts [7]. Although there are several studies that explore the quality of life and mental health disorders among emergency medicine (EM) residents, only a few studies have analyzed gender differences [8]. Gender equality is fundamental for the progression and prosperity of societies. Persistent gender differences in these parameters may affect female physicians disproportionately, making it difficult for them to utilize their abilities and negatively affect the entire EM team. The appropriate development and utilization of qualified female residents are the key to the advancement of the specialty globally.

EM physicians in South Korea, both who are board-certified and during training programs have reported excessive stress, poor sleep quality, and consequential low satisfaction in their specialty [9–11]. Furthermore, Korean female training physicians experienced more depressive symptoms related to occupational stress [12]. Since 2010, the Korean Society of Emergency Medicine (KSEM) conducted a comprehensive survey of board-certified EM physicians every 5 years [13]. After that, longitudinal evaluation and analysis of attending EM physicians can be made. Despite this, research solely focussed on young training EM physicians in residency has not been performed yet.

The KSEM conducted the first survey to evaluate the mental health and quality of life in EM residents. Health risks among the EM residents have not been studied before, and this is the first report of analyzing the survey. Our primary aim was to evaluate the prevalence of depression and sleep–wake disorders among EM residents in South Korea. The secondary aim was to identify gender differences and situations that adversely affect the mental health of the residents and improve the training environment to ensure psychological and emotional support.

## Methods

### Background and study design

**Figure 1.**
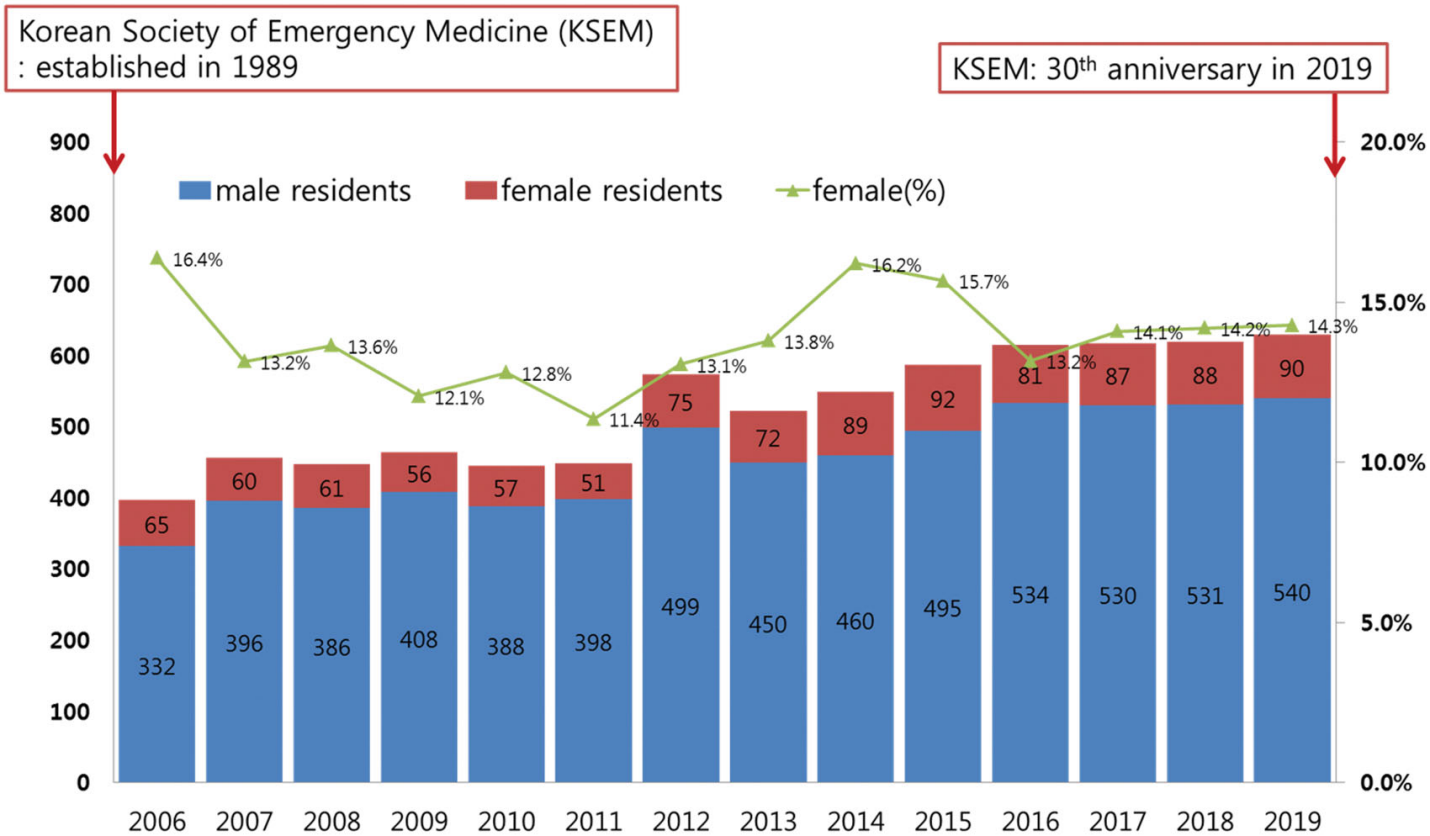
Annual census and gender ratio of emergency medicine residents in South Korea.

It has been 30 years since the KSEM was established in South Korea. The EM residency training course and EM certification began in 1994 and 1997, respectively. In comparison to other specialized medical courses, EM was certified somewhat later in Korea. A total of 98 EM residency programs at each teaching hospital are registered in Korea and every course is unified in a 4-year program under a single organization. We summarized the overall characteristics of the emergency medical system and human resources during the residency training in Figure 1 and Supplementary Table 1. In South Korea, each ED is classified into three categories by its facility and resources. Level 1 Regional Emergency Medical Centre (EMC) is the highest level of ED, which is similar to Level 4 ED in Australia or Type 1 ED in the United Kingdom. Most of Level 1 ED is a fully equipped tertiary referral hospital in the urban area and academic teaching institutions for most sub-specialties. The number of Level 1 ED is regulated by the government according to their location and populations in designated territories, even for nationwide distribution. Level 2 Local EMC is the second level of ED. Local EMC includes general hospitals and community hospitals and is located in both rural and urban areas. Some EDs in large general hospitals are categorized as Level 2 Local EMC based on government policies. Level 3 Local Emergency Medical Facility is the minimum level of ED and usually offers simple emergency care in minor cases.

The KSEM conducted the prospective 2019 National EM Resident Wellness Survey study over a 3-month period from 1 April 2019 to 30 June 2019. We contacted eligible residents by text message to participate in the survey, in addition to individually sending an e-mail to each of the 630 EM residents. The questionnaire was available on the KSEM website for online submission, and each resident had secure access. The participants completed the survey after consenting to its purpose and intent, which was indicated at the start of the survey. A total of 384 responses were received. We treated unanswered items as missing values for the analysis (one respondent did not provide answers regarding history of medication use, and the other provided incomplete answers in the PHQ-9 questionnaire). Data from 382 respondents (response rate: 60.6%) were included in the analysis of this cross-sectional study. The survey organizers performed a quality verification and data confirmation process on the data from which personally identifiable information was deleted. In December 2019, the data was released and made available to the investigators and members of the KSEM society. We obtained approval from the education and research committee of the KSEM, which conducted the first survey on academic EM residents to better understand the current status of mental and physical stress during residency training, as well as the perceived professional challenges or obstacles in South Korea.

### Data collection and variables

We collected demographic data on age, sex, marital status, chronic medical condition, use of caffeine or medications (e.g. antidepressants, sedatives, and sleep aids), subspecialty, level of residency, and on-call duty. A cross-sectional survey was conducted using the self-administered 9-item depression module of the full Patient Health Questionnaire (PHQ-9). The PHQ-9 is a reliable and valid tool, not only to establish the diagnosis of a depressive disorder but also to grade the severity of depressive symptoms [14]. The diagnostic validity of the PHQ has been established [15]. It has a high sensitivity (88%, 95% confidence interval [CI] 83–92%) and specificity (85%, 95% CI 82–88%) for diagnosing major depressive disorders and is considered comparable to clinician-administered assessments. A score of 10 on the PHQ-9 represents the cut-off point for differentiating moderate from severe depression. This study utilized the Epworth Sleepiness Scale (ESS), which was developed to measure daytime sleepiness and facilitate the diagnosis of sleep-wake disorders [16]. A score of 0–10 is considered normal, whereas a score from 11 to 24 indicates excessive daytime sleepiness. For instance, scores of 11–15 indicate mild-to-moderate excessive daytime sleepiness with the possibility of sleep apnoea. A score of 16 and above suggests severe excessive daytime sleepiness that may be associated with narcolepsy [16]. The Apgar Wellness Score (AWS) is a scale designed to assess and reflect physicians’ wellness status [17]. The name “Apgar” was adopted from the neonatal evaluating system (Appearance, Pulse, Grimace, Activity, and Respiration). Similar to the neonate Apgar system, the adult Apgar consists of five questions, and depending on observed conditions, a score of 0–2 is assigned to each. A total AWS of 9–10 indicates the superior wellness status of respondents, while a score of 5 and below indicated poor wellness status. Further plans for mental health support and interventions to improve wellness are recommended for these groups.

### Measurement focus

The primary outcome focus was the prevalence of depression and sleep–wake disorders through the assessment of working and training characteristics during EM residency. The secondary outcome focus was gender differences in depression and sleep–wake disorder rates among EM residents.

### Statistical analysis

We analyzed the data using IBM SPSS Statistics software, version 25 (IBM Corp., Armonk, NY, USA) and MedCalc version 17 (MedCalc Software, Mariakerke, Belgium). Categorical variables were presented as frequencies and percentages, whereas continuous variables were reported as means ± standard deviations (*SD*), or as medians and interquartile ranges (IQRs, 25th–75th percentile). We used the Chi-squared or Fisher’s exact test to compare categorical variables. The normality of the distribution of the variables was determined using the Shapiro–Wilk test. Normally distributed data were expressed as means and standard deviations, while medians and IQRs were used for non-normally distributed data. A *t*-test or one-way analysis of variance and the Mann–Whitney *U*-test were used to compare continuous variables. Using two-way analysis of variance, we assessed the sleepiness and depression score gaps among EM residents across varying residency years, and according to their gender. Univariate and multivariate regression analyses were used to identify risk factors for sleepiness or depression rates among the study participants. The odds ratio (OR) and 95% CI were estimated. All tests were two-tailed, and a *p*-value of <.05 was considered to indicate statistical significance.

## Results

### Characteristics of the study subjects

In 2019, 90 female EM residents were in training nationally (14.3% of the total 630 residents). The gender ratio of the trainees in EM remained at 11–16% for the past several years (Figure 1). Among the total 630 EM residents (90 females, 540 males), 60 of the 90 female residents responded (66.7%), while 322 of the 540 male residents responded (49.5%). We analyzed data from 382 respondents out of a total of 384 survey participants in this study. Overall, 15.7% were female (*n* = 60). The mean age of both males and females was 30 years, and more than one-third of them were married. The majority (71.7%) of female EM residents worked mainly in level 2, whereas 53.1% of the male residents worked in level 1 emergency medical centre (EMC). Table 1 shows the gender gap in the clinical activity and flexibility of shift trade. The mean working hours per week was 68.0 (95% CI 60.0–72.0) with 10 night shifts per month. While 49.1% of the male residents did not report any challenges in changing their working schedule, only ten females (16.7%) reported that they could switch their shift schedule easily (*p <* .001). Almost all the residents consumed caffeinated drinks during overnight duties (*n* = 330, 86.4%), but only a few provided a history of using antidepressants, benzodiazepines, and other narcotics (*n* = 50, 13.1%) or sleep aids (*n* = 67, 17.5%). More than half of the participants (*n* = 212, 55.5%) reported a history of alcohol consumption and male residents demonstrated a significantly higher rate of smoking (*p =* .005, Table 1).

**Table 1.** General characteristics of survey respondents by gender.

	Overall (*n* = 382)	Female resident (*n* = 60, 15.7%)	Male resident (*n* = 322, 84.3%)	*p*-Value*
Age^†^	30.0 (28.0–34.0)	30.0 (28.8–33.3)	30.0 (28.0–34.0)	.603
Marital status				.948
Single	237 (62.0)	37 (61.7)	200 (62.1)	
Married	145 (38.0)	23 (38.3)	122 (37.9)	
Level of residency				.745
R1	94 (24.6)	18 (30.0)	76 (23.6)	
R2	93 (24.3)	14 (23.3)	79 (24.5)	
R3	96 (25.1)	13 (21.7)	83 (25.8)	
R4	99 (25.9)	15 (25.0)	84 (26.1)	
Level of training EMC				*<.001*
Level 1 (regional EMC)	188 (42.9)	17 (28.3)	171 (53.1)	
Level 2 (local EMC) or others	194 (50.8)	43 (71.7)	151 (46.9)	
History of taking medicines				
TCA, BZP, narcotics	50 (13.1)	12 (20.0)	38 (11.8)	.084
Sleep aids, zolpidem	67 (17.5)	12 (20.0)	55 (17.1)	.585
Caffeine during night duty	330 (86.4)	54 (90.0)	276 (85.7)	.374
History of smoking	87 (22.8)	4 (6.7)	83 (25.8)	*.005*
History of alcohol	212 (55.5)	26 (43.3)	186 (57.8)	.114
Clinical activity^†^				
Weekly worked hours, total	68.0 (60.0–72.0)	72.0 (58.0–72.0)	64.0 (60.0–75.0)	.773
Monthly night duty/night shift	10.0 (8.0–12.3)	11.0 (8.8–13.0)	10.0 (8.0–12.0)	.057
Flexibility of shift trade				*.009*
Yes	270 (70.7)	34 (56.7)	236 (73.3)	
No	112 (29.3)	26 (43.3)	86 (26.7)	
Depression and life quality^†^				
PHQ-9 depression score	6.0 (3.0–10.0)	7.0 (3.8–12.0)	5.0 (3.0–10.0)	*.004*
Epworth sleepiness scale	8.0 (6.0–12.0)	11.5 (7.0–14.0)	8.0 (6.0–12.0)	*.015*
Apgar wellness score	5.0 (4.0–8.0)	5.0 (4.0–7.0)	5.0 (4.0–8.0)	*.026*

Unless otherwise indicated, data are reported as number (%) of emergency physicians.

*p‐Values reflect Mann–Whitney *U* tests and chi‐square comparisons where appropriate.

^†^Data are reported as median (interquartile range, 25–75th percentile).

### Depression and sleep–wake disorders among EM residents

**Figure 2.**
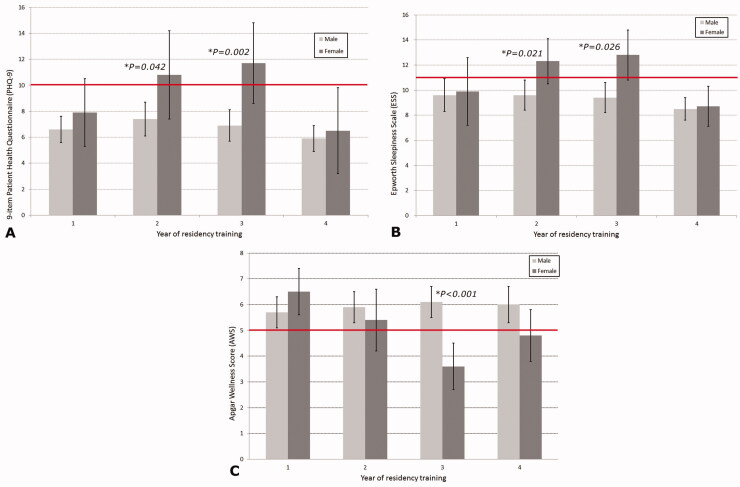
Gender differences in Patient Health Questionnaire (PHQ-9), Epworth sleepiness scale (ESS), and Apgar wellness scores (AWS) according to residency years. (A) Gender differences in the PHQ-9 score. For depression symptoms, PHQ-9 is >10 points. (B) Gender differences in the ESS score. An ESS score of 11 suggests the presence of excessive sleepiness. (C) Gender differences in the AWS. Individuals with AWS <5 points need professional counselling for significant trouble or pain.

Based on the PHQ-9, 27.5% of the EM residents met the criteria for at least moderate depression (PHQ-9 score ≥ 10) (Table 2). Marital status and the post-graduate year of the residents did not show significant differences (*p* = .167 and *p* = .144, respectively). In contrast to the significant effect of the frequent night shifts on depression (*p =* .005, OR 1.930), working for more or <70 h per week was not a significant risk factor (*p* = .849). In residents who could trade their shift schedule easily, risk factors for depression decreased significantly (*p =* .001, OR 2.22). The PHQ-9 and ESS scores were significantly higher in female residents (*p* = .004 and *p* = .015, respectively) and the AWS also showed a significant difference between males and females (*p* = .026) (Table 1). Both male and female residents reported low PHQ-9 scores during the first year of training, and no significant difference was observed. However, in the second and third years of training, while the PHQ-9 scores of male residents were ≤10, the scores of female residents increased significantly (*p* = .042 and *p* = .002, respectively). Similar results were obtained when using the ESS, with the second-year resident level (R2) and third-year resident level (R3) females reporting scores >11, indicating the presence of excessive daytime sleepiness and demonstrating a significant difference when compared to male respondents (*p* = .021, *p* = .026). Among the years of residency, third-year female residents scored under 5 in the AWS, showing a significant difference when compared to male residents of the same year (*p <* .001). Compared to male colleagues within the same residency years, female EM residents of R2 and R3 levels worked similar weekly hours and monthly night shifts; however, they scored higher in the PHQ-9 and ESS scales (Figure 2 and Table 3). With regards to sleep-related problems, 36.9% of the EM residents had excessive daytime sleepiness (ESS ≥ 11 points, Table 4).

**Table 2.** Logistic regression of predictors for depression among the survey participants.

	Grouping of variables	Depression (PHQ-9 ≥ 10) *n* = 105 (27.5%)	No Depression (PHQ-9 < 10) *n* = 277 (72.5%)	Univariate Crude Odds Ratio (95% CI)	*p*-Value	Multivariable Adjusted Odds Ratio (95% CI)	*p*-Value*
Gender	Male	82 (78.1)	240 (86.6)	Reference		1	
	Female	23 (21.9)	37 (13.4)	1.82 (1.02–3.24)	*.042*	1.95 (1.05–3.60)	*.034*
Age group	<30 years old	39 (37.1)	100 (36.1)	Reference		1	
	≥30 years old	66 (62.9)	177 (63.9)	0.96 (0.60–1.52)	.850	1.06 (0.64–1.77)	.801
Marital status	Single	71 (67.6)	166 (59.9)	Reference		1	
	Married	34 (32.4)	111 (40.1)	0.72 (0.45–1.15)	.167	0.87 (0.51–1.48)	.611
Level of residency	R1	24 (22.9)	70 (25.3)	1.46 (0.71–2.87)	.315	1.33 (0.62–2.85)	.460
	R2	32 (30.5)	61 (22.0)	2.07 (1.08–3.97)	*.028*	2.00 (0.99–4.04)	.053
	R3	29 (27.6)	67 (24.2)	1.71 (0.89–3.29)	.109	1.66 (0.83–3.31)	.149
	R4	20 (19.0)	79 (28.5)	Reference		1	
Training EMC	Level 1	58 (55.2)	130 (46.9)	Reference		1	
	Level 2, others	47 (44.8)	147 (53.1)	0.73 (0.46–1.15)	.175	0.60 (0.37–0.96)	*.035*
Weekly hours worked	<70 h	58 (55.2)	156 (56.3)	Reference		1	
	≥70 h	47 (44.8)	121 (43.7)	1.05 (0.67–1.64)	.849	0.68 (0.40–1.16)	.158
Monthly night duty/shift	Night duty <11	54 (51.4)	186 (97.1)	Reference		1	
	Night duty ≥11	51 (48.6)	91 (32.9)	1.93 (1.22–3.05)	*.005*	1.97 (1.17–3.31)	*.010*
Flexibility of shift trade	Yes	61 (58.1)	209 (75.5)	Reference		1	
	No	44 (41.9)	68 (24.5)	2.22 (1.38–3.56)	*.001*	2.12 (1.25–3.60)	*.005*

^†^Adjusted by gender, age group, marital status, level of residency and training hospital, and working strength (weekly hours and monthly night duty).

**Table 3.** Depression, daytime sleepiness, and wellness scores in emergency medicine residents according to gender and residency training years.

	R1			R2			R3			R4		
	Female	Male	*p*-Value*	Female	Male	*p*-Value*	Female	Male	*p*-Value*	Female	Male	*p*-Value*
Age	29.3 ± 2.1	29.0 ± 3.0	.349	30.1 ± 4.1	29.9 ± 3.1	.824	31.8 ± 1.6	31.8 ± 3.2	.956	33.2 ± 4.0	32.3 ± 2.9	.511
Hours worked, weekly	75.6 ± 10.3	70.9 ± 11.8	.419	62.9 ± 12.0	69.0 ± 0.7	.095	62.2 ± 8.9	64.7 ± 11.5	.442	62.1 ± 11.9	57.0 ± 11.2	.145
Night shift, monthly	10.2 ± 3.2	10.9 ± 3.3	.328	11.7 ± 1.9	10.9 ± 3.4	.280	10.7 ± 1.6	10.0 ± 3.1	.424	10.2 ± 4.8	8.7 ± 3.1	.015*
PHQ-9	7.9 ± 5.4	6.6 ± 4.6	.355	10.8 ± 6.0	7.4 ± 6.1	.042*	11.7 ± 5.3	6.9 ± 5.0	.002*	6.5 ± 5.9	5.9 ± 4.7	.822
Epworth sleepiness scale	9.9 ± 6.0	9.6 ± 5.7	.806	12.3 ± 3.3	9.6 ± 5.1	.021*	12.8 ± 3.1	9.4 ± 5.2	.026*	8.7 ± 2.7	8.5 ± 3.9	.697
Apgar wellness score	6.5 ± 1.8	5.7 ± 2.2	.094	5.4 ± 2.3	5.9 ± 2.3	.391	3.6 ± 1.4	6.1 ± 2.3	<.001*	4.8 ± 1.8	6.0 ± 2.6	.133

Unless otherwise indicated, data are reported as mean ± *SD* (standard deviation).

*p‐Values reflect Mann–Whitney *U* tests and two-way ANOVA where appropriate.

**Table 4.** Risk factors associated with excessive daytime sleepiness among the emergency medicine residents.

Variables overall (*N* = 382)	Grouping of variables	Daytime sleepiness (Epworth scale ≥11) *n* = 141 (36.9%)	No daytime sleepiness (Epworth scale < 11) *n* = 241 (63.1%)	Crude OR (95% CI)	*p*-Value	Adjusted OR (95% CI)	*p*-Value*
Gender	Male	111 (34.5)	211 (65.5)	Reference		1	
	Female	30 (50.0)	30 (50.0)	1.90 (1.09–3.31)	*.024*	1.81 (1.01–3.26)	*.049*
Level of residency	R1	33 (36.3)	58 (63.7)	1.88 (0.99–3.54)	*.050*	1.94 (0.93–4.06)	.078
	R2	44 (47.3)	49 (52.7)	2.97 (1.59–5.51)	*.001*	3.13 (1.57–6.22)	*.001*
	R3	39 (40.6)	57 (59.4)	2.26 (1.22–4.20)	*.010*	2.44 (1.26–4.71)	*.008*
	R4	23 (23.2)	76 (76.8)	Reference		1	

^†^Adjusted for training hospital, working strength (weekly hours and monthly night duty), and flexibility of shift trading. To determine the logistic model calibration, we calculated the Hosmer-Lemeshow goodness of fit (*p* = .819). Data are represented as number (row %).

### Disparity in the prevalence of sleepiness, quality of life, and depression

Female EM residents showed a higher prevalence of depression and excessive daytime sleepiness compared to their male colleagues (OR 1.95, 95% CI: 1.05–3.60; OR 1.81, 95% CI: 1.01–3.26, respectively; Tables 2 and 4). Logistic regression analysis revealed significant gender differences in depression (OR 1.95, *p* = .034), flexibility of trading shift (OR 2.12, *p* = .005), level of training EMC (OR 0.60, *p* = .035), and frequent night shifts (OR 1.97, *p* = .010). However, age (OR 1.06, *p* = .801), marital status (OR 0.87, *p* = .611), and working above the average hours (OR 0.68, *p* = .158) were not significantly associated with depression of sleep-wake disorders.

## Discussion

To the best of our knowledge, this is the first cross-sectional study to examine gender differences in depression and sleep-related problems among EM residents in Korea. In total, 27.5% of EM residents met the criteria for depression and 36.9% reported excessive daytime sleepiness. The risk factors for depression included shift trade flexibility and frequent night shifts. Female EM residents had higher PHQ-9 and ESS scores, with lower AWS than male EM residents. Moreover, female EM residents had a higher risk of depression (adjusted odds ratio [AOR] = 1.95, 95% CI: 1.05–3.60, *p* = .034) and daytime sleepiness (AOR = 1.81, 95% CI: 1.01–3.26, *p* = .049) compared to their male colleagues. In females, the associated risk factors for depression included shift trade flexibility, level of training EMC, and frequent night shifts.

Over 25% of EM residents were affected by depression, and an even greater proportion reported sleep-wake disorders. Compared to previous studies, these figures are considered notable [2,8]. The unique characteristics of EM, including irregular work schedules and highly extensive workloads, could attribute to the substantial findings of this study. Working at night can adversely affect the circadian rhythm [18]. Several studies have found that sleep deprivation and poor sleep quality from prolonged night shifts are associated with fatigue, lack of concentration, memory problems, and depression in physicians [6,19–21]. Burnout experienced by training emergency physicians could be another significant factor of interest in explaining these inter-relationships [22]. Compared to other departments, the increase in clerical burden and interaction with other consultants in the ED could be related to burnout. Furthermore, EM reported the highest burnout rate among other medical specialties [23].

In our study, female EM residents showed higher PHQ-9 and ESS scores, which demonstrates a greater prevalence of depression and excessive daytime sleepiness compared to their male colleagues. Moreover, lower AWS in female EM residents could be indicative of having to face more stressful circumstances than male EM residents. Depression and sleep problems among female medical residents were also reported in comparable studies [19,20,24,25]. Gender equality has been a major societal topic in South Korea for decades. Despite this, there are still several demanding societal roles viewed as implicitly feminine, regardless of whether females work in demanding fields, such as medicine [26]. Balancing career development, motherhood, and family responsibilities continue to challenge female physicians worldwide and could be a potential source of extensive stress [27,28]. As such, gender bias can be a substantial factor contributing to stress among female physicians. Nevertheless, EM is generally considered a male-dominated specialty with a majority of EM physicians being male in South Korea [29]. Leadership positions are traditionally held by senior clinicians. Gender bias, both explicit and implicit, can present barriers to enter both academic seniority as well as positions of authority for female physicians [29].

In our research, depression and excessive daytime sleepiness were more prevalent among females compared to male EM residents. Furthermore, the flexibility of shift trade, level of training EMC, and frequent night shifts were significant predictors of depression severity. The association between depressive symptoms and the difference in the registered emergency centre, with the shift trade flexibility, is one of the most interesting findings of our study. In South Korea, academic institutions with abundant resources mainly consist of level 1 ED (188 residents at 36 sites) in contrast to the greater number of level 2 ED (194 residents at 118 sites) that are community-based hospitals. In the field of academic medicine, regardless of specialties, pronounced gender gaps still exist and hinder promotion [30]. Over the male-dominant nature of the specialty, disparities in academic promotion for females are additionally evident with the existing discrepancy of attending physicians in level 1 centres. In this regard, we hypothesize that female applicants tend to avoid upscale emergency centres due to the perceived lack of female role models or the so-called “old-boy” atmosphere present in many male-dominated environments [31]. In addition, characteristics of higher-level emergency centres, such as high work demands with excessive physical and emotional intensity, may also support their decision [32]. Paradoxically, contrary to their expectations, small faculties with limited manpower and fewer resources at lower-level institutions tend to restrict the flexibility of their duties. Persistent poor sleep quality, accompanied by loaded or hectic night shifts, are established factors for a variety of health problems [19]. According to our study, the frequency of night shifts was associated with depression. Despite this, 10 night shift duties per month can still be a burden, even for young physicians. In contrast, <5 monthly night shifts were reported in several countries [33].

In previous research, excessive working hours or academic training years have been reported as significant risk factors for both physical and psychological problems [6,33]. Our study demonstrated a low correlation between the number of working hours and depression. Medical residents usually work for long hours, and it is frequently due to their lower position as trainees in the medical hierarchy [34]. In this study, the mean working hours were 68 h per week. These are relatively fewer hours compared to other studies and worldwide recommendations of suitable working hours [35]. We presume that working within standard limits is an important factor for these results. Previous studies have revealed a strong connection between working hours and depression in case of extremely long working hours (over 100 h per week) [6,33]. Furthermore, we found that academic training years did not have a linear relation to depression, and this finding is comparable to the results of another study [8]. Interestingly, female physicians in the middle year of their training had the highest risk of depression. In contrast to a previous study, which reported a higher rate of depressive symptoms at the beginning of training, our study showed a progressive increase in PHQ-9 and ESS scores in female residents every year until their third year of training [33]. As female trainees become more senior, their roles in the hospital naturally increase, and consequently, the burdens experienced both at home and in the workplace can have an adverse influence on their mental health. Compared to junior residents, senior physicians usually face more responsibilities regarding clinical decisions or procedures and manage more critical patients, resulting in chronic exposure to stressful environments [22,36].

Wellness and work-life balance are important issues, regardless of the level of training or even profession. As our study has shown, female EM residents in particular face greater threats to their mental health. The implementation of a nationwide modification of the training system should be discussed to address health problems and improve overall wellbeing. In 2019, the Korean Ministry of Health approved the partial amendment of the medical resident training program. Thereafter, medical residents who are currently involved in ED cannot exceed the maximum of three night shifts per week. In addition, the duration of the off-duty period between shift works must be longer than working hours. As a result of one trial in paediatric residents, a positive influence on their performance was achieved after limiting the duration of shift hours [21]. Further research should be followed to evaluate the health problems in medical residents after the implementation of these policies. In addition, providing more flexibility in work schedules may help reduce the associated risk in female EM residents. Preventive strategies that reduce work-family conflict should be designed and implemented. In other countries, several studies have been conducted to improve the circumstances of residents and to improve their quality of life [37]. These studies have reported a substantial improvement in the training environment, especially a significant improvement in PHQ-9 and ESS scores, and life satisfaction among physicians [38,39]. Thus, the positive outcomes from these studies should be considered in future discussions.

Our study has several limitations. First, the low response rate may have led to a bias. It is possible that the director of specific institutions, who were more confident about their training environment, may encourage their trainees to participate in the survey. If that was the case, the results in our study underestimated the existing gender disparity. Thus, the study findings should be interpreted with caution. Since many emergency physicians did not respond, the bias was possibly enhanced by skewing the responses towards certain academic hospitals or geographic regions. Second, findings may have been affected by the relatively small sample size that may have overestimated the prevalence and severity of depression. Third, although it may differ from one training site to another, a more detailed survey on shift schedules like the duration of each work, presence of rotating, or even information on colleagues working in shifts together may be helpful in analyzing present problems. Other limitations are related to the nature of self-reporting data and lack of gold standard diagnostic clinical interviews. This could have also skewed the results, along with the possible under-representation of the prevalence of depression and taking tricyclic antidepressants/antipsychotics/sedatives. More detailed surveys should be conducted to examine the relationships of depression and sleep-wake disorders with family members or the presence of own child, as family responsibilities are an important factor for gender differences. Therefore, the responses analyzed in this study are only representative of the individuals who participated, and the data may not be generalizable to all female emergency residents in Korea. Thus, the acquisition of additional data regarding the participation and career perceptions of female residents is pivotal to further understand this subject.

## Conclusions

We found that a significant gender gap exists between male and female EM residents with respect to the prevalence of, and risk factors for, depression and sleep-wake disorders. Female residents in particular demonstrated crucial problems with overall wellbeing compared to their male colleagues. In line with the unique findings of shift trade flexibility and frequent night shifts practically affecting entire EM residents, results of female residents were related to the level of training site and this could be an exceptional discovery of our study. Differences in work stress and exposure between male and female residents must be considered for the prevention and treatment of depression during EM residency training. To alleviate these risk factors, our findings suggest that it is necessary to consider several modifications to potentially enhance the training environment and start the wellness program run by the KSEM.

## Supplementary Material

Supplemental Material

## Data Availability

The data that support the findings of this study are available on request from the corresponding author, WY. The data are not publicly available because that could compromise the privacy of research participants.

## References

[1] World Health Organization. Depression. A global public health concern. Developed by Marina Marcus, M. Taghi Yasamy, Mark van Ommeren, and Dan Chisholm, Shekhar Saxena. WHO Department of Mental Health and Substance Abuse [cited 2021 Jan 25]. https://www.who.int/mental_health/management/depression/who_paper_depression_wfmh_2012.pdf

[2] Mata DA, Ramos MA, Bansal N, et al. Prevalence of depression and depressive symptoms among resident physicians: a systematic review and meta-analysis. JAMA. 2015;2373–2383. 10.1001/jama.2015.15845 PMC4866499 26647259

[3] Fahrenkopf AM, Sectish TC, Barger LK, et al. Rates of medication errors among depressed and burnt out residents: prospective cohort study. BMJ. 2008;336(7642):488–491. 18258931 10.1136/bmj.39469.763218.BE PMC2258399

[4] Druss BG, Rosenheck RA, Sledge WH. Health and disability costs of depressive illness in a major U.S. corporation. Am J Psychiatry. 2000;157(8):1274–1278. 10910790 10.1176/appi.ajp.157.8.1274

[5] Takagi K, Tagami T. Work-style reform of emergency physicians: the Japanese experience. Eur J Emerg Med. 2019;26(6):398–399. 31688220 10.1097/MEJ.0000000000000640

[6] Kim JH, Yoon J, Kim SS. Association between long working hours and depressive symptoms among interns and residents in South Korea-2014 Korea interns & residents survey. J Korean Soc Occup Environ Hyg. 2015;25(2):236–243.

[7] Guille C, Frank E, Zhao Z, et al. Work-family conflict and the sex difference in depression among training physicians. JAMA Intern Med. 2017;177(12):1766–1772. 29084311 10.1001/jamainternmed.2017.5138 PMC5820732

[8] Katz ED, Sharp L, Ferguson E. Depression among emergency medicine residents over an academic year. Acad Emerg Med. 2006;13(3):284–287. 16495424 10.1197/j.aem.2005.10.009

[9] Kim YG, Ryoo HW, Seo KS, et al. Job stress, job satisfaction of emergency residents and its related factors. J Korean Soc Emerg Med. 2008;19(6):749–759.

[10] Kim JK, Kim YJ, Seo KS, et al. Job stress, job satisfaction and occupational commitment among Korean emergency physicians. J Korean Soc Emerg Med. 2010;21(2):246–258.

[11] Kim HB, Park KH, Kim I, et al. Self-perceived quality of sleep in Korean emergency physicians. J Korean Soc Emerg Med. 2010;27(5):67–75.

[12] Kim K, Lee S, Choi YH. Relationship between occupational stress and depressive mood among interns and residents in a tertiary hospital, Seoul, Korea. Clin Exp Emerg Med. 2015;2(2):117–122. 27752582 10.15441/ceem.15.002 PMC5052868

[13] Lee HM, Cho KH, Yang HJ, et al. 2010 Korean society of emergency physician survey. J Korean Soc Emerg Med. 2014;25:238–251.

[14] Muñoz-Navarro R, Cano-Vindel A, Medrano LA, et al. Utility of the PHQ-9 to identify major depressive disorder in adult patients in Spanish Primary Care Centres. BMC Psychiatry. 2017;17(1):291. 28793892 10.1186/s12888-017-1450-8 PMC5550940

[15] Levis B, Benedetti A, Thombs BD, et al. Accuracy of patient health questionnaire-9 (PHQ-9) for screening to detect major depression: individual participant data meta-analysis. BMJ. 2019;365:l1476. 30967483 10.1136/bmj.l1476 PMC6454318

[16] Johns MW. A new method for measuring daytime sleepiness: the Epworth Sleepiness Scale. Sleep. 1991;14(6):540–545. 1798888 10.1093/sleep/14.6.540

[17] Bintliff S. The adult APGAR: a guide to physician wellness. Int J Dermatol. 2012;51(7):868–869. 22715837 10.1111/j.1365-4632.2011.05378.x

[18] Kervezee L, Shechter A, Boivin DB. Impact of shift work on the circadian timing system and health in women. Sleep Med Clin. 2018;13(3):295–306. 30098749 10.1016/j.jsmc.2018.04.003

[19] Jaradat R, Lahlouh A, Mustafa M. Sleep quality and health related problems of shift work among resident physicians: a cross-sectional study. Sleep Med. 2020;66:201–206. 31978863 10.1016/j.sleep.2019.11.1258

[20] Goebert D, Thompson D, Takeshita J, et al. Depressive symptoms in medical students and residents: a multischool study. Acad Med. 2009;84(2):236–241. 19174678 10.1097/ACM.0b013e31819391bb

[21] Rahman SA, Sullivan JP, Barger LK, et al. Extended work shifts and neurobehavioral performance in resident-physicians. Pediatrics. 2021;147(3):e2020009936. 33619044 10.1542/peds.2020-009936 PMC7919117

[22] Lin M, Battaglioli N, Melamed M, et al. High prevalence of burnout among US emergency medicine residents: results from the 2017 national emergency medicine wellness survey. Ann Emerg Med. 2019;74(5):682–690. 30879701 10.1016/j.annemergmed.2019.01.037

[23] Shanafelt TD, Boone S, Tan L, et al. Burnout and satisfaction with work-life balance among US physicians relative to the general US population. Arch Intern Med. 2012;172(18):1377–1385. 22911330 10.1001/archinternmed.2012.3199

[24] Wada K, Yoshikawa T, Goto T, et al. National survey of the association of depressive symptoms with the number of off duty and on-call, and sleep hours among physicians working in Japanese hospitals: a cross sectional study. BMC Public Health. 2010;10:127. 20222990 10.1186/1471-2458-10-127 PMC2848631

[25] Peterlini M, Tibério IF, Saadeh A, et al. Anxiety and depression in the first year of medical residency training. Med Educ. 2002;36(1):66–72. 11849526 10.1046/j.1365-2923.2002.01104.x

[26] Lorello GR, Silver JK, Moineau G, et al. Trends in representation of female applicants and matriculants in Canadian residency programs across specialties, 1995 to 2019. JAMA Netw Open. 2020;3(11):e2027938. 33231640 10.1001/jamanetworkopen.2020.27938 PMC7686870

[27] Gjerberg E. Women doctors in Norway: the challenging balance between career and family life. Soc Sci Med. 2003;57(7):1327–1341. 12899913 10.1016/s0277-9536(02)00513-0

[28] Nomura K, Yamazaki Y, Gruppen LD, et al. The difficulty of professional continuation among female doctors in Japan: a qualitative study of alumnae of 13 medical schools in Japan. BMJ Open. 2015;5(3):e005845. 10.1136/bmjopen-2014-005845 PMC4386232 25818268

[29] Hansen M, Schoonover A, Skarica B, et al. Implicit gender bias among US resident physicians. BMC Med Educ. 2019;19(1):396. 31660944 10.1186/s12909-019-1818-1 PMC6819402

[30] Silver JK. Understanding and addressing gender equity for women in neurology. Neurology. 2019;93(12):538–549. 31366723 10.1212/WNL.0000000000008022

[31] Saalwachter AR, Freischlag JA, Sawyer RG, et al. The training needs and priorities of male and female surgeons and their trainees. J Am Coll Surg. 2005;201(2):199–205. 16038816 10.1016/j.jamcollsurg.2005.03.016

[32] Basu S, Qayyum H, Mason S. Occupational stress in the ED: a systematic literature review. Emerg Med J. 2017;34(7):441–447. 27729392 10.1136/emermed-2016-205827

[33] Ogawa R, Seo E, Maeno T, et al. The relationship between long working hours and depression among first-year residents in Japan. BMC Med Educ. 2018;18(1):50. 29587738 10.1186/s12909-018-1171-9 PMC5870810

[34] Busireddy KR, Miller JA, Ellison K, et al. Efficacy of interventions to reduce resident physician burnout: a systematic review. J Grad Med Educ. 2017;9(3):294–301. 28638506 10.4300/JGME-D-16-00372.1 PMC5476377

[35] Accreditation Council for Graduate Medical Education. Common program requirement [cited 2021 Jan 31]. https://www.acgme.org/What-We-Do/Accreditation/Common-Program-Requirements

[36] Vanyo L, Sorge R, Chen A, et al. Posttraumatic stress disorder in emergency medicine residents. Ann Emerg Med. 2017;70(6):898–903. 28826753 10.1016/j.annemergmed.2017.07.010

[37] Mache S, Bernburg M, Baresi L, et al. Mental health promotion for junior physicians working in emergency medicine: evaluation of a pilot study. Eur J Emerg Med. 2018;25(3):191–198. 27879536 10.1097/MEJ.0000000000000434

[38] Kersemaekers WM, Vreeling K, Verweij H, et al. Effectiveness and feasibility of a mindful leadership course for medical specialists: a pilot study. BMC Med Educ. 2020;20(1):34. 32019524 10.1186/s12909-020-1948-5 PMC7001198

[39] Spiotta AM, Fargen KM, Patel S, et al. Impact of a residency-integrated wellness program on resident mental health, sleepiness, and quality of life. Neurosurgery. 2019;84(2):341–346. 30169852 10.1093/neuros/nyy112

