# Supplementary material for: High prevalence of depression and sleep–wake disorders among female emergency medicine residents in South Korea
Source: Ann Med. 2022 Mar 29;54(1):846–55. doi: 10.1080/07853890.2022.2053568 (PMC8967212; doi:10.1080/07853890.2022.2053568)
Supplement: Supplemental Material [file IANN_A_2053568_SM9168.docx]

**Supplement A.** System and human resources of emergency medicine system in South Korea between Year 2016 and Year 2019.

|  | **Year 2016** | **Year 2019** |
| --- | --- | --- |
| **Human Resources** |  |  |
| Board-certified Emergency Physicians (EP), Total | 1,418 | 1,843 |
| Female EP, n (%) | 170 (12.0) | 234 (12.7) |
| EP density per 100,000 national population | 2.7 | 3.6 |
| ED Residency training | 587 | 630 |
| Female residents, n (%) | 92 (15.7) | 90 (14.3) |
| Resident density per 100,000 national population | 1.1 | 1.2 |
| Board-certified EMT, Total |  |  |
| EMT-intermediate | 5,047 | 9,512 |
| EMT-basic | 3,997 | 9,170 |
| **Resources of EMS System** |  |  |
| EMS Facility, Total | 546 | 521 |
| Level 1 (Regional emergency medical center) | 20 (3.7) | 36 (6.9) |
| Level 2 (Local EMC) | 126 (23.1) | 118 (22.6) |
| Level 3 (Local emergency facility) | 274 (50.2) | 248 (47.6) |
| Other emergency room or facilities | 126 (23.1) | 119 (22.8) |
| Number of beds in EMS facilities, total | 7,099 | 6,945 |
| Annual census of ER visits count | 8,259,966 | 8,749,305 |
| ER census per each certified EP | 8,268 | 6,540 |
| ER census per each bed of emergency facility | 1,192 | 1,260 |

EP; emergency physician, ED; emergency department, ER; emergency room, EMS; emergency medicine system, EMT; emergency medical technician.
